# Burnout, psychopathology and purpose in life in healthcare workers during COVID-19 pandemic

**DOI:** 10.3389/fpubh.2022.926328

**Published:** 2022-08-05

**Authors:** Marcelo O'Higgins, Luz Angela Rojas, Iván Echeverria, Lorena Roselló-Jiménez, Ana Benito, Gonzalo Haro

**Affiliations:** ^1^Department of Psychiatry, School of Medical Sciences, National University of Asunción, Asunción, Paraguay; ^2^Fundación Universitaria del Área Andina, Bogotá, Colombia; ^3^Department of Mental Health, Consorcio Hospitalario Provincial de Castellón, Castellón de la Plana, Spain; ^4^TXP Research Group, Universidad Cardenal Herrera-CEU, CEU Universities, Castellón de la Plana, Spain; ^5^Department of Basic Psychology, Clinic and Psychobiology, Jaume I University, Castellón de la Plana, Spain; ^6^Torrente Mental Health Unit, Hospital General de Valencia, Torrente, Spain

**Keywords:** acute stress, anxiety, burnout, COVID-19, depression, healthcare workers, psychopathology, purpose in life

## Abstract

**Background:**

The COVID-19 pandemic has led to a significant increase in the workload of healthcare workers that, together with the risks associated with exposure to this new virus, has affected their mental health.

**Objectives:**

The objective of the current study was to determine the prevalence of psychopathology and burnout syndrome in healthcare workers and the predictive role of purpose in life and moral courage in this relationship.

**Methods:**

A cross-sectional study was carried out in 115 Spanish healthcare workers.

**Results:**

Participants with burnout had higher anxiety (*p* = 0.001), depression (*p* < 0.001), post-traumatic stress (*p* = 0.01) and alcohol consumption (*p* = 0.03) levels. The different components of burnout (emotional fatigue and despersonalization) were associated with the occurrence of anxiety (OR = 0.31) and depression (OR = 0.26), respectively. A strong purpose in life decreased emotional fatigue (OR = −0.39) depersonalization (OR = −0.23) scores, increased personal accomplishment (OR = 0.52), subsequently reducing burnout levels (OR = −0.45).

**Conclusions:**

Purpose in life was most strongly related to decreased levels of burnout. Furthermore, an association between anxiety, depression and the components of burnout was found.

## Introduction

Since the start of the COVID-19 pandemic in March 2020, health systems worldwide have experienced levels of demand that, in many cases, have exceeded the care capacity for which they were designed (1). This translated into a significant increase in the workloads and job demands put on healthcare workers which, together with exposure to this unknown infectious agent, impacted their mental health (2). Previous work has already described how the inadequate availability of devices and resources to deal with the pandemic in care institutions, care work involving continuous exposure to COVID-19, increased working hours, concern about work-life balance, and the risk of exposing loved ones because of their work, negatively affected the mental health of healthcare workers (3–5). To date, multiple studies have been published on the psychopathologies healthcare professionals have developed in relation to the COVID-19 pandemic, observing an increase in the rates of anxiety, depression, and post-traumatic stress, especially in healthcare workers on the front line (6–8).

It is possible that the work requirements, together with the risk of acquiring the disease, can lead healthcare professionals to develop burnout syndrome (9) as a response to interpersonal and work stressors (10). Previous studies carried out in Spanish healthcare workers during the early stages of the pandemic showed medium to high levels of burnout syndrome in this population (11). Different scales have been designed to measure burnout as a construct, such as the *Maslach Burnout Inventory—Human Services Survey* (MBI-HSS), which has three subscales that assess emotional exhaustion, depersonalization, and personal accomplishment (12). Likewise, various studies have reported the relationship between high levels of burnout and the development of anxiety, depression, and post-traumatic stress disorder in healthcare professionals during the COVID-19 pandemic (13). However, certain characteristics of these healthcare workers, such as purpose in life (PIL) and moral courage (MC), could be involved in the impact burnout syndrome has on the mental health of these professionals.

On the one hand, PIL is defined as the perception that an individual has about the purpose and value of their life, playing a guiding role in life goals and in making decisions about the use of personal resources (14, 15). Indeed, high levels of PIL in healthcare workers who faced the COVID-19 pandemic were associated with a lower prevalence of psychopathology (16). On the other hand, MC is the ability to face danger or social disapproval when performing what one believes to be their duty (17). Paradoxically, not being able to act in accordance with these moral values can generate psychopathology through the concept of moral distress, as has been seen during the COVID-19 pandemic (16). This was particularly true in the early stages of the pandemic when healthcare professionals had to decide whether to put their lives at risk to save those of others or when they had to assess which patients received care and which could not be cared for due to a lack of resources.

Although some studies have already evaluated the relationship between burnout syndrome and the development of psychopathology in healthcare workers during the COVID-19 pandemic, given all the above, constructs such as PIL and MC could still be helpful in understanding this relationship. Therefore, the objective of this study was to determine the prevalence of psychopathology and burnout syndrome in healthcare workers and the predictive role PIL and MC might have in this context. We hypothesized that (a) healthcare workers had a high prevalence of mental disorders as well as burnout syndrome during the COVID-19 pandemic; (b) the presence of psychopathology in healthcare workers is related to the extent of the burnout syndrome; and (c) PIL and MC are factors that can predict the relationship between psychopathology and burnout syndrome.

## Methods

### Study design and participants

This was a cross-sectional observational study which followed the STROBE guidelines for observational studies. A total of 115 Spanish healthcare workers were recruited using a snowball strategy, focusing on recruiting all the healthcare personnel in the Provincial Consortium Hospital of Castellon (Spain) (*n* = 97), the second largest hospital in the city, and in other dependent Department of Health centers in Castellon (*n* = 18) between 20 September and 18 November 2021, the dates delimiting the two highest peaks of COVID-19 contagion in Spain. This hospital is responsible for mental health care in the province of Castellon [included in this study 38 health providers with 14 nurses (36.8%), 10 doctors (26.3%), 10 nurse auxiliary technicians (26.3%), 2 psychologist (5.2%), 1 occupational therapists (2.6%) and 1 administrators (2.6%)], ICU [included in this study 10 health providers with 5 nurses (50%), 3 nurse auxiliary technicians (30%) and 2 doctors (20%)], and Internal Medicine [included in this study 7 health providers with 4 doctors (57.1%), 2 nurses (28.5%), 1 nurse auxiliary technicians (14.2%)] among other specialties including Ophthalmology (*n* = 5), Emergency Department (*n* = 4), Oncology (*n* = 2) Preventive medicine (*n* = 2), Digestive Medicine (*n* = 1), and other departments (*n* = 29).

To calculate the sample size we used G^*^Power software (v3.1.9.4) (18) to calculate that a sample size of 96 would be required when considering an expected effect size of *d* = 0.55, an alpha of 5%, and beta of 20% for 2 groups with an allocation ratio of 1.8, when performing Mann–Whitney *U*-tests.

### Variable measurements

After signing the informed consent, the healthcare workers who participated in the study completed a series of self-administered instruments in Spanish, including a questionnaire of sociodemographic variables that asked about age, sex, religiosity, marital status, occupation, history of physical illness and mental disorders, addictions, and variations in mental health compared to the start of the pandemic. All these instruments were validated for Spanish speakers.

To assess anxiety, depression, and post-traumatic stress disorder (PTSD) we used the *Beck Anxiety Inventory* [BAI; cut-off point (CP) = 8] (19), *Beck Depression Inventory* (BDI-II; CP = 14) (20), and the Diagnostic and Statistical Manual of Mental Disorders (DSM-5) criteria for post-traumatic stress disorder (PTSD), respectively. Drug abuse was assessed using the *Drug Abuse Screening Test* (DAST-10; CP = 1) (21) and alcohol abuse was tested using the *Alcohol Use Disorders Identification Test* (AUDIT; CP for women = 6, CP for men = 8) (22). Total scores and dichotomous variables were calculated to divide the participants into a group of individuals whose score exceeded the scale of the CPs and a group that did not.

Purpose in life was analyzed using the *Purpose in Life scale* (PIL; CP = 113) (23), calculating a dichotomous variable to differentiate between individuals who had a sense of PIL and those who did not. Moral courage was assessed with the *Moral Courage Scale for Physicians* (MCSP) (24) and the *Professional Moral Courage Scale* (PMCS) (25). To evaluate the presence of burnout syndrome, the Spanish version of the MBI-HSS was used, applying a CP for each subscale: ≥27 for emotional exhaustion, ≥10 for depersonalization and ≤33 for personal fulfillment (12).

### Data analysis

SPSS software (version 23) for Microsoft (IBM Corp., Armonk, NY) was used for all the statistical analyses. After the exploratory and descriptive study, the quantitative variables were compared using Mann–Whitney *U*-tests because the data presented asymmetry. The categorical variables were compared using Pearson chi-squared tests. After verifying that the statistical assumptions were met, logistic and linear regression models were created to predict the presence of burnout, the total burnout score, and the extent of emotional exhaustion, depersonalization, and personal fulfillment. Finally, the data were modeled using the PROCESS add-on (v3.4) for SPSS (26) to test how the variables studied were related to each other.

The ethical principles set out in the Declaration of Helsinki and the Council of Europe Convention were followed and the informed consent of all participants was obtained. Moreover, data confidentiality was guaranteed according to the General Data Protection Regulation (GDPR; 2018). This study was authorized by the Investigation Commission at the Provincial Hospital Consortium in Castellon (ref. A-15/04/20) and the Clinical Research Ethics Committee at the Cardenal Herrera-CEU University (ref. CEI20/068).

## Results

### Sociodemographic characteristics and the presence of psychopathology in the sample cohort

For the total of 115 Spanish healthcare workers evaluated, the median age was 42 years, and the majority were female (65.2%, *n* = 75). Regarding occupation, 35.8% (*n* = 39) were nursing staff, 29.4% (*n* = 32) were physicians, and 13.8% (*n* = 15) were auxiliary nursing care technicians. In relation to their marital status, the majority were married (55%, *n* = 64). All 115 healthcare workers were vaccinated.

In relation to psychopathology, 47.8% (*n* = 55) presented at least one mental disorder among those evaluated in this study; 36.5% presented anxiety (*n* = 42), 15.7% depression (*n* = 18), and 21.7% met the criteria for post-traumatic stress disorder (*n* = 25). There were no differences in the presence of these disorders among the different professional categories (at least one mental disorder: χ^2^ = 3.55, *p* = 0.31; anxiety: χ^2^ = 6.82, *p* = 0.07; depression: χ^2^ = 4.81, *p* = 0.18; post-traumatic stress disorder: χ^2^ = 7.32, *p* = 0.06). There were also no differences between the younger and older participants (at least one mental disorder: χ^2^ = 3.35, *p* = 0.06; anxiety: χ^2^ = 0.23, *p* = 0.62; depression: χ^2^ = 0.38, *p* = 0.53; post-traumatic stress disorder: χ^2^ = 0.01, *p* = 0.91). Only 6.1% (*n* = 7) of the participants were previously in psychological/psychiatric treatment and they accounted for only 9.1% (*n* = 5) of those with at least one disorder.

Regarding the variation in mental health status compared to the first year of the COVID-19 pandemic, 31.3% (*n* = 36) reported worsening, 45.2% (*n* = 52) reported no change, and 20% reported having improved (*n* = 23). 15.7% (*n* = 18) of the sample were smokers. Considering the consumption of other substances, according to the AUDIT and DAST-10, we observed that 7.8% (*n* = 9) and 8.7% (*n* = 10) presented risky consumption of alcohol and drugs, respectively. Of the total sample, 38.53% (*n* = 42) of the participants exceeded the CP in at least one of the burnout syndrome dimensions. Specifically, 19.3% (*n* = 21) exceeded the emotional exhaustion CP, 30.3% (*n* = 33) the depersonalization CP, and 15.7% (*n* = 18) the personal accomplishment CP.

### The sociodemographic characteristics, purpose in life, moral courage, and psychopathological variables of the groups according to the presence or absence of burnout

Table 1 shows the sociodemographic characteristics of the participants according to the presence of burnout. Both groups differed in their marital status, with a higher percentage of singles in the group with burnout (45.2%) compared to the group without burnout in which the majority were married (65.7%; χ^2^ = 8.37, *p* = 0.03). Occupation was not associated with the presence of burnout (χ^2^ = 12.09, *p* = 0.17). No significant differences were found for the rest of the sociodemographic variables.

**Table 1:** Sociodemographic characteristics of the study participants according to the presence of burnout and differences between the study cohort groups.

**Variables**	**Total** ***n* = 115** **% (*n*)/Median (IQR)**	**With burnout ** ***n* = 42** **% (*n*)/Median (IQR)**	**Without burnout ** ***n* = 67** **% (*n*)/Median (IQR)**	**χ^2^/Mann-Whitney U** ** test (*p*)**
**Age**	42 (21)	41 (24)	43 (20)	549.5 (0.486)
**Sex**				
Female	65.2% (75)	56.1% (23)	72.7% (48)	3.13 (0.077)
Male	32.2% (37)	43.9% (18)	27.3% (18)	
**Religiosity yes**	53% (61)	43.9% (18)	63.1% (41)	3.74 (0.053)
**Marital status**				
Single	33% (38)	**45.2% (19)**	22.4% (15)	**8.37 (0.039)**
Married	55% (64)	45.2% (19)	**65.7% (44)**	
Divorced	9.6% (11)	7.1% (3)	11.9% (8)	
Widowed	0.9% (1)	2.4% (1)	0% (0)	
**Physical illness yes**	2.6% (3)	2.4% (1)	1.6% (1)	0.092 (0.76)
**Smoker yes**	15.7% (18)	14.6% (6)	18.5% (12)	0.26 (0.60)
**History of addiction yes**	0% (0)	0% (0)	0% (0)	
**History of mental disorder yes**	9 (7.8%)	9.5% (4)	7.7% (5)	0.11 (0.73)

*The groups from among the categorical variables in which the corrected typified residuals were significant (less than −1.96 or greater than 1.96) are shown in bold. n, sample; IQR, Interquartile range; χ^2^, Pearson chi-squared test*.

Table 2 shows the differences in the psychopathological variables of the participants according to the presence of burnout. The group without burnout showed a higher score for PIL (Me = 116. IQR = 16) than the burnout group (Me = 102, IQR = 26). When the CP of the PIL scale was used, the group without burnout presented a higher percentage of high PIL (62.7%; *n* = 42) than the group with burnout (28.6%; *n* = 12) (χ^2^ = 12.02, *p* = 0.001). Participants without burnout more often reported that their mental health had remained unchanged or had even improved, although the difference between the groups was not significant (χ^2^ = 4.12, *p* = 0.12).

**Table 2:** Scores for moral courage, purpose in life, and psychopathological variables according to the presence of burnout and differences between the cohort groups.

**Variables**	**Total ** ***n* = 115** **% (*n*)/Median (IQR)**	**With burnout ** ***n* = 42** **% (*n*)/Median (IQR)**	**Without burnout ** ***n* = 67** **% (*n*)/Median (IQR)**	**χ^2^/Mann-Whitney U** ** test (*p*)**
**Mental health variation**				4.12 (0.12)
Worsened	31.3% (36)	45% (18)	26.2% (17)	
Improved	20% (23)	20% (8)	23.1% (15)	
No variation	45.2% (52)	35% (14)	50.8% (33)	
**MCSP**	8 (2)	8 (2)	8 (2)	1,270.5 (0.772)
**PMCS**	11 (1)	10 (2)	11 (2)	1,081 (0.077)
**PIL**	112 (24)	102 (26)	**116 (16)**	**741.5 (<0.001)**
**PIL yes**	47.8% (55)	28.6% (12)	**62.7% (42)**	**12.02 (0.001)**
**BAI**	5 (10)	**10.5 (13.2)**	3 (7)	**837 (0.001)**
**Anxiety yes**	36.5% (42)	**54.8% (23)**	26.9% (18)	**8.56 (0.003)**
**BDI-II**	5 (9)	**10 (9.75)**	3 (5)	**618 (<0.001)**
**Depression yes**	15.7% (18)	**31% (13)**	7.5% (5)	**10.33 (0.001)**
**PTSD**	4 (6.25)	**5.5 (8.25)**	2 (6)	**951 (0.011)**
**PTSD Yes**	21.7% (25)	**33.3% (14)**	16.4% (11)	**4.17 (0.0041)**
**AUDIT**	2 (3)	**3 (2.5)**	2 (2)	**1,014.5 (0.031)**
**Alcohol yes**	7.8% (9)	9.5% (4)	7.5% (5)	0.14 (0.70)
**DAST-10**	0 (0)	0 (0)	0 (0)	1,205 (0.067)
**Drugs yes**	8.7% (10)	14.3% (6)	6% (4)	2.14 (0.14)
**Psychopathology yes**	47.8% (55)	**66.7% (28)**	38.8% (26)	**8.01 (0.005)**

*The groups from among the categorical variables in which the corrected typified residuals were significant (less than −1.96 or greater than 1.96) are shown in bold; n, sample; IQR, Interquartile range; χ^2^, Pearson chi-squared test; MCSP, Moral Courage Scale for Physicians; PMCS, Professional Moral Courage Scale; PIL, Purpose In Life; BAI, Beck Anxiety Inventory; BDI-II, Beck Depression Inventory-II; PTSD, Post-Traumatic Stress Disorder; DAST-10, Drug Abuse Screening Test; AUDIT, Alcohol Use Disorders Identification Test*.

We observed a global increase in psychopathology in the group of participants with burnout (66.7%, *n* = 28) compared to the group without burnout (38.8%, *n* = 26) (χ^2^ = 8.01, *p* = 0.005). Moreover, the group with burnout presented a higher score in the BAI (*p* = 0.001) and a higher percentage of participants with anxiety (χ^2^ = 8.56, *p* = 0.003). The burnout group presented a higher score in the BDI-II (*p* < 0.001) and there were more participants with depression (χ^2^ = 10.33, *p* = 0.001). Likewise, the group of participants with burnout had a higher PTSD score (*p* = 0.01) and showed a higher percentage of PTSD (χ^2^ = 4.17, *p* = 0.004).

### Binary and linear logistic regressions and data modeling

Table 3 shows the variables that allowed burnout and its dimensions to be predicted. High PIL scores were associated with a decreased probability of burnout [OR = −0.45; 95% CI (−0.80, −0.36); *p* < 0.001], emotional fatigue [OR = −0.39; 95% CI (−0.42, −0.15); *p* < 0.001], and depersonalization [OR = −0.23; 95% CI (−0.15, −0.006); *p* = 0.034], and were associated with an increased probability of presenting personal fulfillment [OR = 0.52; 95% CI (0.14, 0.27); *p* < 0.001]. High BAI scores were associated with an increased likelihood of burnout [OR = 0.26; 95% CI (0.18, 0.87); *p* = 0.003] and emotional fatigue [OR = 0.31; 95% CI (0.14, 0.55); *p* = 0.001], and high scores on the BDI-II were associated with an increased likelihood of depersonalization [OR = 0.26; 95% CI (0.03, 0.33); *p* = 0.01]. Finally, being married was associated with an increased probability of presenting personal fulfillment [OR = 0.16; 95% CI (0.01, 3.39); *p* = 0.04].

**Table 3:** Significant odds ratios from logistic and linear regression models predicting burnout.

**Response**	**Predictors**	**Odds ratio/beta (95% confidence interval)**	***P*-value**
**Presence of burnout**	Purpose in life	0.94 (0.91, 0.97)	<0.001
**Total burnout**	Purpose in life	−0.45 (−0.80, −0.36)	<0.001
	Beck Anxiety Inventory	0.26 (0.18, 0.87)	0.003
**Emotional exhaustion**	Purpose in life	−0.39 (−0.42, −0.15)	<0.001
	Beck Anxiety Inventory	0.31 (0.14, 0.55)	0.001
**Depersonalization**	Purpose in life	−0.23 (−0.15, −0.006)	0.034
	Beck Depression Inventory-II	0.26 (0.03, 0.33)	0.018
**Personal fulfillment**	Purpose in life	0.52 (0.14, 0.27)	<0.001
	Marital status	0.16 (0.01, 3.39)	0.048

**Figure 1:**
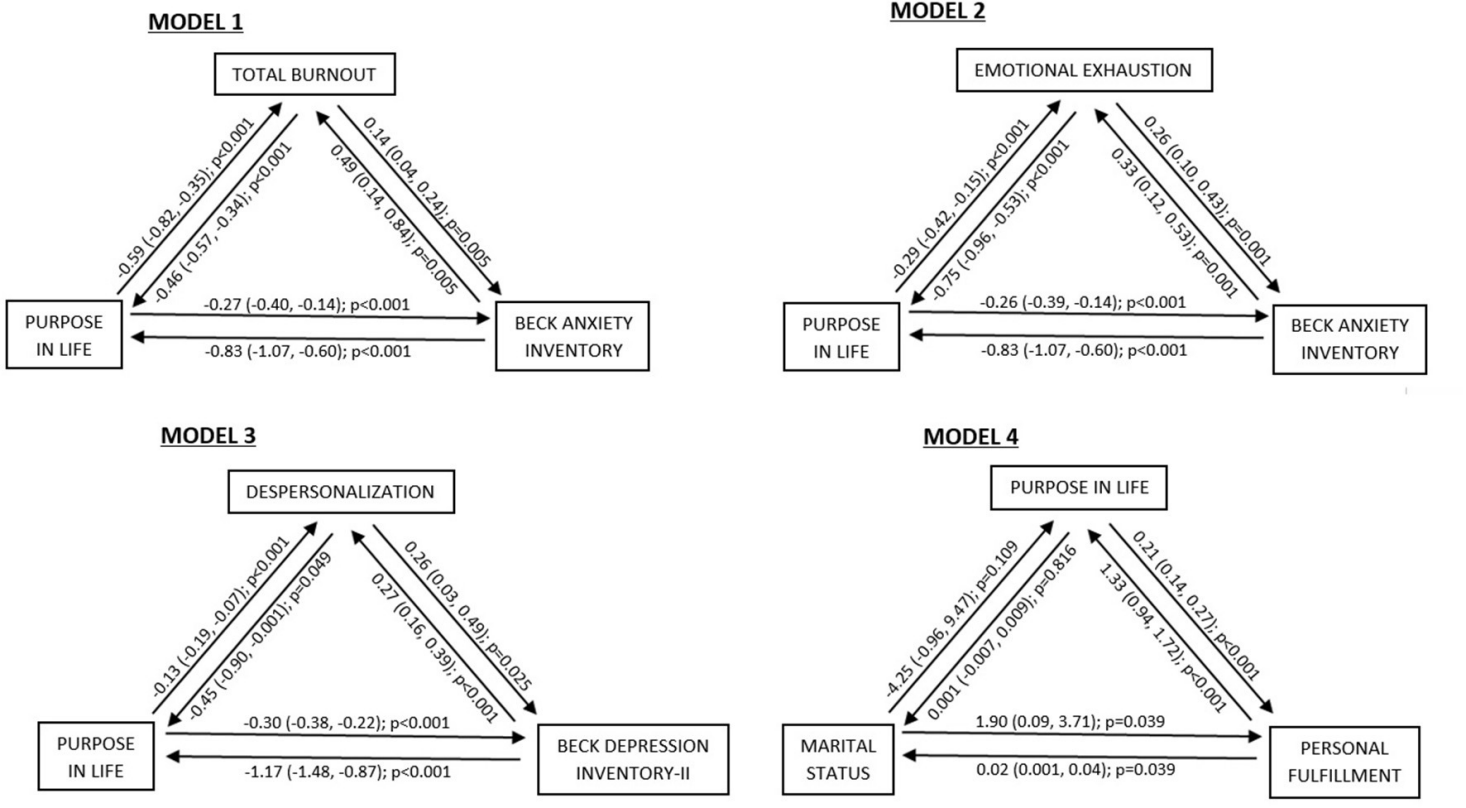
Exploratory models of psychopathology in healthcare workers.

We modeled the data according to the results obtained in the logistic regressions and the models with the best fit are shown in Figure 1. No moderation or mediation effects were found in models 1 (total burnout, PIL, and BAI), 2 (emotional exhaustion, PIL, and BAI), or 3 (depersonalization, PIL, and BDI-II), although these three variables did influence each other. In model 4 we found that personal fulfillment had a reciprocal influence on both marital status and PIL, while marital status and PIL were not related to each other.

## Discussion

When considering the results in the healthcare workers participating in this study, we observed that the prevalence of burnout (38.53%) was lower than the average reported in a recent systematic review in which a prevalence of 54% was reported during the COVID-19 pandemic (27). Similarly, a Canadian study found a 51.8% prevalence of burnout, with at least 1 day of symptoms of emotional exhaustion and depersonalization (28). However, in a study conducted in Japan where a prevalence of 50% was found in workers caring for patients with COVID-19 in the early stages of the pandemic, in subsequent measurements burnout had reduced to 30%. This figure was related to the decrease in the number of patients with COVID-19 being treated and later increased to 43.1% in a new epidemiological wave of the virus (29). The figure reported during this inter-peak period is similar to that found in our study and could be explained by the lower number of cases of COVID-19 infections at the time of data collection. Therefore, the prevalence of burnout was still high and was consistent with the figures previously reported in other studies. Another study comparing different waves of the pandemic found an increase in measures of anxiety, depression and stress between two consecutive waves (30). It could also be speculated that burnout could be maintained over time or even worsen in subsequent waves if appropriate measures are not taken to intervene in maintaining factors affecting health providers.

For global psychopathology, we found a prevalence of 47.8% in our healthcare workers, compared to a study in Canada with a similar population in which a prevalence of 35.6% had been reported. Of these, 24.3% presented PTSD, 23.3% anxiety, and 10.6% depression (28). In our study, the figures for PTSD and depression were similar to those reported by Cyr et al. (28), but were higher for anxiety. A similar study in Ireland found higher prevalence values for depression with a prevalence between 22 and 28% in the general population during the pandemic (31). It should also be noted that in countries such as Brazil, India, and the United States, variation was observed in the figures for depression in relation to the moment the data were collected during the pandemic, where an increase was presented during the peaks of COVID-19 contagion (32). A series of cases of psychotic depression in healthcare workers during the pandemic showed that a number of factors could influence the development of a mental disorder in at-risk populations, in this case, healthcare workers with significant pandemic-related stress (33). This stressful context could also have influenced the participants in the current study.

Regarding emotional exhaustion, depersonalization, and lack of personal fulfillment, figures of 19.3%, 30.3%, and 15.7%, respectively we report here were lower compared to the figures of 51%, 52%, and 21% reported by Ghahramani et al. (27). They were also very similar to a study conducted in Libya which reported 67.1% emotional exhaustion, 47.4% depersonalization, and 22.7% for lack of accomplishment (34). In this aforementioned study, the fear of COVID-19 was associated with high scores in the dimensions of emotional exhaustion and depersonalization, while being older than 35 years was associated with higher figures for depersonalization (34). This was also observed in a study conducted in Poland, in which participants with more than 20 years of experience reported higher scores in all three dimensions of the scale (35).

The logistic regressions we carried out showed that PIL was most consistently related to burnout, with the total PIL score and the score for its emotional exhaustion, depersonalization, and personal fulfillment dimensions representing predictors of the presence of burnout. Some previous studies that sought to relate PIL to the presence of psychopathology indicated that high levels of PIL were related to a reduction in the appearance of symptoms of anxiety and depression (36–38). PIL has also been linked to lower levels of hopelessness and worry in patients with eating disorders (39). Additionally, BAI has also been shown to be a predictor of burnout. In this sense, a study conducted in Italy during the COVID-19 pandemic found a significant association between higher levels of anxiety and burnout, especially the emotional exhaustion dimension of burnout (40).

Likewise, the logistic regressions indicated that the presence of depersonalization was associated with high BDI-II scores, which agrees with previous studies indicating a relationship between depersonalization and symptomatology of depression and anxiety (41). Other studies have also previously related the presence of the symptoms of depression and depersonalization with other components of burnout syndrome such as emotional exhaustion (42). Moreover, marital status was also associated with personal accomplishment in this current work, which is consistent with a study conducted in Korea which found that marital status, gender, workload related to the care of patients with COVID-19, and the presence of the symptoms of anxiety and depression were able to predict emotional exhaustion. This same study indicated that profession, job satisfaction, anxiety, and depression were also related to high levels of depersonalization (43).

Other studies have highlighted the role that PIL could have as a mediator factor in the mental health of healthcare workers during the COVID-19 pandemic (15), indicating that higher levels of PIL could act as a protective factor for the mental health of these workers. Indeed, the positive perspectives of the present and the past could moderate PIL, allowing individuals to experience a greater degree of resilience and better perspectives for the future, which would be very useful when devising ways to face the difficulties derived from the pandemic (43). However, a study conducted on adolescents reported that PIL failed to moderate the relationship between the impact of the COVID-19 pandemic and depressive symptoms, anxiety, sleep problems, and behavioral disturbances (44).

Higher levels of PIL are related to increased levels of personal accomplishment, therefore explaining why lower levels of burnout are found in some healthcare professionals. In this sense, a study in China reported moderate levels of compassion satisfaction (that is, wellbeing at work) and low levels of burnout in nursing healthcare professionals, which was explained by the presence of professional collaboration, a sense of solidarity, opportunities for professional growth, and awareness of professional purpose in these participants (45). These results are similar to other work in Spanish caregivers, which reported lower levels of burnout and higher levels of compassion satisfaction in nurses compared to medical staff (11). Therefore, the PIL could be useful for the construction of screening instruments or for the design of psychotherapeutic interventions aimed at enhancing individual mental health (46).

One way to address the increased symptoms of anxiety and depression in healthcare workers in order to reduce the burnout experienced and to increase the purpose in life of these people could be to use mindfulness-based techniques to reduce work-related stress and improve psychological wellbeing (47). Other techniques that have already been used with healthcare workers with favorable results were Yoga (48) and Tai Chi (49) sessions seeking to reduce anxiety symptoms and improving sleep quality. Protocols for teaching emotional regulation techniques to healthcare personnel have also been tried and have shown efficacy in their initial applications, so further research on these aspects is needed (50).

The main limitation of this current work was its cross-sectional design which prevented us from inferring any causal relationships between the metrics. The models obtained in this study did not show mediation or moderation effects but rather, a reciprocal influence between the variables and so longitudinal studies would be required to clarify these relationships. Another possible limitation of this work was that some healthcare professionals did not agree to participate due to the length of the questionnaires we used. However, studies that included questionnaires with different lengths reported that the length of the questionnaire and the number of items included had not affected the measurement parameters of the constructs or quality of the data obtained (51). No data were extracted from participants on job satisfaction and the frequency and number of shifts they made during the pandemic. This could have important implications for the development of burnout symptoms. Another limitation that can be noted is the determination of a clear exposure to patients with COVID-19. By the time this study was conducted, the medical center where the study was conducted had already gone through several waves of cases within the context of the COVID-19 pandemic. Most of them had already been exposed to working with COVID-19 patients, however, having a clear measure of exposure would be useful to compare those who treated COVID-19 patients with those who did not. It might even be of interest to see if there are differences between practitioners depending on the number of COVID-19 patients treated by the different practitioners. These aspects would be of interest to be addressed in future similar studies.

## Conclusions

The PIL metric was most consistently related to low burnout in this work and so it could be an object of interest in future work aiming to prevent psychopathology, including burnout syndrome, in healthcare personnel. Additionally, the logistic regressions we performed demonstrated an association between anxiety, depression, marital status, and the components of burnout syndrome.

## Data availability statement

The raw data supporting the conclusions of this article will be made available by the authors, without undue reservation.

## Ethics statement

The studies involving human participants were reviewed and approved by Investigation Commission at the Provincial Hospital Consortium in Castellon (ref. A-15/04/20) and the Clinical Research Ethics Committee at the Cardenal Herrera-CEU University (ref. CEI20/068). The patients/participants provided their written informed consent to participate in this study.

## Author contributions

MO'H, LR, and LR-J: recruitment of participants, data collection, and writing of original draft. IE: conceptualization, recruitment of participants, review, and editing of manuscript. AB: conceptualization, formal analysis, and supervision. GH: conceptualization, supervision, and funding acquisition. All authors approved the contributions, read, and approved the final manuscript.

## Funding

This study was received funding from the Universidad Cardenal Herrera–CEU, CEU Universities (FUSP-PPC-19-7CF9E6DA; Grant INDI21/29) and Fundación de Investigación del Hospital Provincial de Castelló (CAF 22–05; 22–06). The funder was not involved in the study design, collection, analysis, interpretation of data, the writing of this article, and the decision to submit it for publication.

## Conflict of interest

The authors declare that the research was conducted in the absence of any commercial or financial relationships that could be construed as a potential conflict of interest. The reviewer IG-M declared a shared affiliation, though no other collaboration, with one of the authors LR-J to the handling Editor.

## Publisher's note

All claims expressed in this article are solely those of the authors and do not necessarily represent those of their affiliated organizations, or those of the publisher, the editors and the reviewers. Any product that may be evaluated in this article, or claim that may be made by its manufacturer, is not guaranteed or endorsed by the publisher.
